# Serological evidence of infection with dengue and Zika viruses in horses on French Pacific Islands

**DOI:** 10.1371/journal.pntd.0007162

**Published:** 2019-02-07

**Authors:** Cécile Beck, Isabelle Leparc-Goffart, Denise Desoutter, Estelle Debergé, Hervé Bichet, Steeve Lowenski, Marine Dumarest, Gaelle Gonzalez, Camille Migné, Jessica Vanhomwegen, Stéphan Zientara, Benoit Durand, Sylvie Lecollinet

**Affiliations:** 1 UMR 1161 Virology, ANSES, INRA, ENVA, ANSES Animal Health Laboratory, EURL for equine diseases, Maisons-Alfort, France; 2 Institut de Recherche Biomédicale des Armées, Unité de Virologie—CNR des Arbovirus, Marseille, France; 3 UMR UVE Unité des Virus Emergents, Aix-Marseille Université - IRD 190—Inserm 1207—IHU Méditerranée Infection, Marseille, France; 4 Service des Laboratoires Officiels Vétérinaires Agroalimentaires et Phytosanitaires de Nouvelle-Calédonie, Direction des Affaires Vétérinaires Alimentaires et Rurales de Nouvelle-Calédonie, Païta, New Caledonia; 5 Service du développement rural, Présidence de la Polynésie française, Papeete, Tahiti, French Polynesia; 6 Environment and Infectious Risks Research and Expertise Unit, Department of Infections and Epidemiology, Institut Pasteur, Paris, France; 7 Epidemiology unit, Paris-Est University, ANSES Animal Health Laboratory, Maisons-Alfort, France; University of Texas Medical Branch, UNITED STATES

## Abstract

New Caledonia and French Polynesia are areas in which arboviruses circulate extensively. A large serological survey among horses from New Caledonia and French Polynesia was carried out to investigate the seroprevalence of flaviviruses in the horse population. Here, 293 equine sera samples were screened for flaviviruses using a competitive enzyme-linked immunosorbent assay (cELISA). The positive sera were then confirmed using a flavivirus-specific microsphere immunoassay (MIA) and seroneutralization tests. This serosurvey showed that 16.6% (27/163) and 30.8% (40/130) of horses were positive for cELISA tests in New Caledonia and French Polynesia, respectively, but the MIA technique, targeting only flaviviruses causing neuro-invasive infections in humans and horses (i.e. West Nile virus [WNV], Japanese encephalitis virus [JEV] and tick-borne encephalitis virus [TBEV]), showed negative results for more than 85% (57/67) of the cELISA-positive animals. Seroneutralization tests with the main flaviviruses circulating in the South Pacific revealed that 6.1% (10/163; confidence interval [95% CI] 3.0%-11.0%) of sera in New Caledonia and 7.7% (10/130; 95% CI 3.8%-13.7%) in French Polynesia were positive for dengue virus serotype 1 (DENV1) and 4.3% (7/163; 95% CI 1.7%-8.6%) in New Caledonia and 15.4% (20/130, 95% CI 9.7%-22.8%) in French Polynesia were found positive for Zika virus (ZIKV). Seroprevalence of the JEV and WNV flaviviruses on the 293 samples from both island groups were comparatively much lower (less than 2%). This seroprevalence study in the horse population shows that horses can be infected with dengue and Zika viruses and that these infections lead to seroconversions in horses. The consequences of these infections in horses and their role in ZIKV and DENV epidemiological cycles are two issues that deserve further investigation.

## Author summary

New Caledonia and French Polynesia, located in the South Pacific, are facing circulation of dengue virus (DENV) for a long time and emergence of Zika virus (ZIKV) since 2013. A large serosurvey among horses’ population from these two islands was carried out to investigate the seroprevalence of the main flaviviruses circulating in the South Pacific. We find out that 6 to 7% of equine sera tested were positive for DENV serotype 1 in the two islands and 4% and 15% were positive for ZIKV in New Caledonia and French Polynesia respectively. Our study highlighted serological evidence of DENV serotype 1 and ZIKV infections of horses leading to meaningful seroconversion. Seroprevalence of other mosquito-borne flaviviruses (i.e. Japanese encephalitis and West-Nile viruses) were comparatively much lower (less than 2%) in New Caledonia and French Polynesia groups suggesting the absence of past active circulation of these viruses in both islands. This finding emphasized the need to investigate the consequences of such infections in the horse population and to determine the role of domestic animals in ZIKV and DENV epidemiological cycles.

## Introduction

French overseas territories located in the South Pacific include New Caledonia, French Polynesia and Wallis and Futuna. New Caledonia is composed of one main island and several archipelagoes, and French Polynesia is composed of five archipelagoes (Society, Marquesas, Tuamotu, Gambier and Austral islands).

Many arthropod-borne viruses (arboviruses) circulate in the Pacific Islands, with a series of epidemics caused by the four serotypes of dengue virus (DENV) documented during the last 50 years, and the presence of emerging arboviruses such as Zika virus (ZIKV), which first occurred in the Yap Islands (Federated States of Micronesia) in 2007 or chikungunya virus (CHIKV) reported since 2012 [1, 2].

DENV, a flavivirus that causes a disease with varying descriptions–ranging from asymptomatic infection to hemorrhagic dengue fever sometimes progressing to a shock syndrome–is the most prevalent arbovirus infection in humans in tropical and subtropical countries. Viruses belonging to the *dengue virus* species are classified in four distinct serotypes (DENV1-DENV4) and infection with one viral serotype does not provide protection against the other three [3]. DENV has been circulating for a long time in the Pacific region with sporadic or rare epidemic outbreaks. Its circulation is characterized by waves of one dominant serotype [4, 5]. In 2013, for the first time, all four serotypes were circulating in the Pacific Islands [6]. In New Caledonia, an important outbreak recorded in 2013 was due to the DENV1 serotype [1]; in French Polynesia, not only DENV1, but also DENV3 (which had not been reported since 1996 in the South Pacific) reemerged and spread during the 2013–2017 period for DENV1 and in 2013 for DENV3 [7, 8].

Conversely, ZIKV, another flavivirus involving mild symptoms similar to those caused by DENV, was not present in New Caledonia or French Polynesia until October 2013 when the virus emerged in French Polynesia and caused a large outbreak [9, 10]. In New Caledonia, the first autochthonous cases were reported by mid-January 2014 due to cases imported from French Polynesia to New Caledonia in late November 2013 and subsequently spread extensively in late August 2014 [11].

Other globally spreading mosquito-borne flaviviruses can circulate in the Pacific Islands. West Nile virus (WNV) is present in Asia and considered enzootic in Australia (Kunjin virus, lineage 1b of WNV), whereas Japanese encephalitis virus (JEV) causes the most common mosquito-transmitted encephalitic disease in Asian countries, its presence stretching from Oceania to northern Australia [12–14]. Nevertheless, in New Caledonia and French Polynesia, no active circulation of these two neurotropic flaviviruses has been documented [15, 16].

In New Caledonia and French Polynesia, DENV and ZIKV are transmitted to humans by mosquitoes belonging to genus *Aedes*, with *Aedes aegypti* present in most Pacific Islands as well as local mosquito species, such as *Ae*. *polynesiensis* in French Polynesia [1].

Humans are the primary amplification host for DENV and ZIKV. However, female mosquitoes–even the highly anthropophilic *Ae*. *aegypti* thriving in human-occupied habitats, such as urban areas–can feed on other vertebrate hosts, and anti-ZIKV antibodies have been detected in several domestic species such as horses, goats and cows [17–20].

A large serological survey conducted in horses from New Caledonia and French Polynesia with a competitive enzyme-linked immunosorbent assay (cELISA) revealed positive sera against flaviviruses. Our study sought to (1) identify the flaviviruses, including DENV and ZIKV, circulating in the horse population and causing this seroconversion, (2) compare horse seroprevalence in New Caledonia and French Polynesia, and finally (3) identify possible risk factors associated with this flavivirus seropositivity.

## Methods

### Animal samples

Two equine serological serosurveys were carried out in September 2015 and April 2016 in New Caledonia and French Polynesia, respectively, to determine the seroprevalence of flaviviruses in the equine population. The serological samples were collected once with the kind support of the chief veterinary officers from the two territories (Dr Denise Desoutter and Dr Hervé Bichet, chief veterinary officers of New Caledonia and French Polynesia respectively). Our research was performed in accordance with an approved French Institutional Animal Care and Use protocol during surveillance studies.

**Fig 1:**
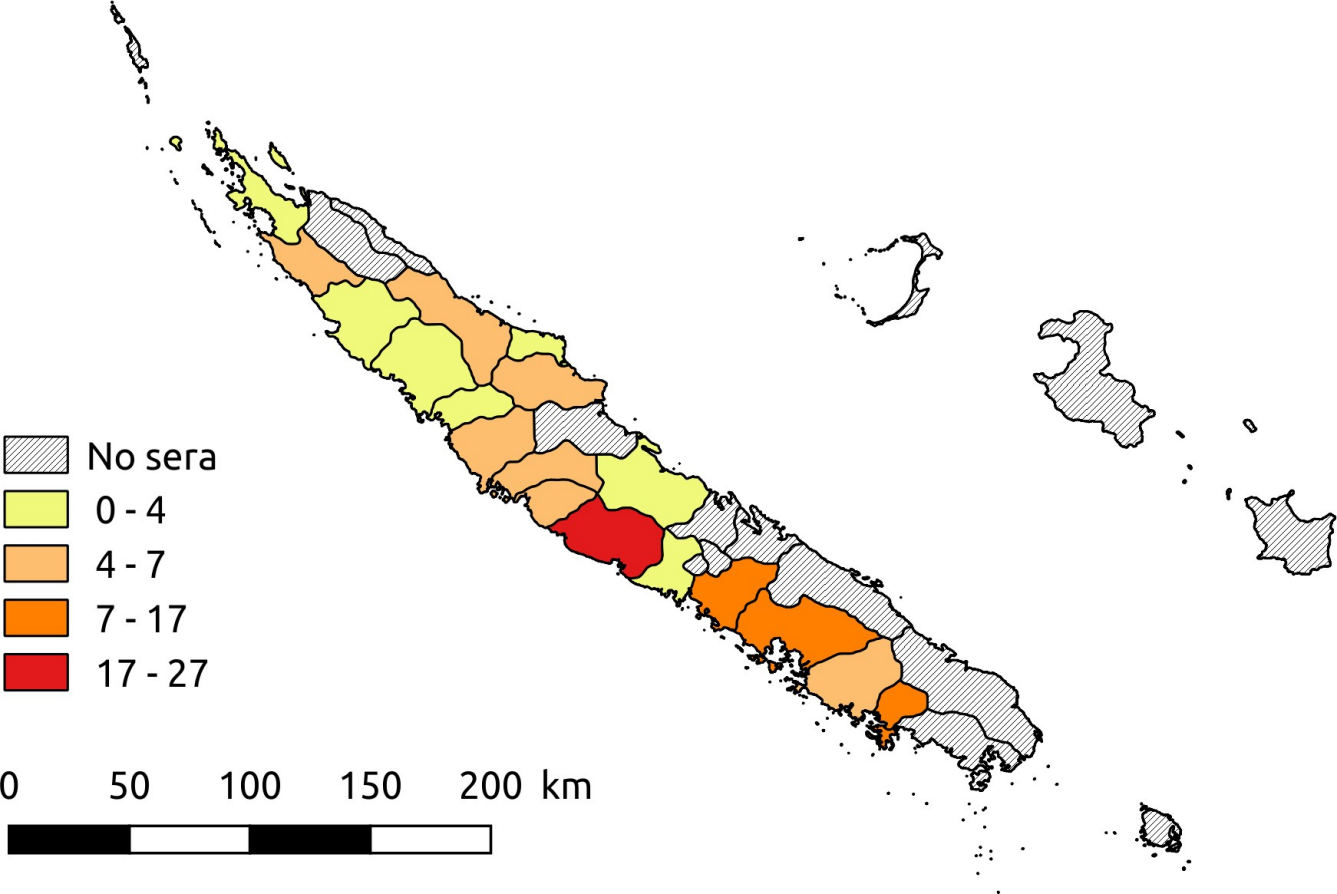
Geographic distribution of horse serum samples collected in New Caledonia (Software: Quantum GIS version 2.18).

In New Caledonia, the 163 equines of the study were located in 20 municipalities distributed across different parts of the main island (Fig 1). In French Polynesia, the survey was conducted on the Marquesas Islands (24 in Nuku Hiva and 27 in Hiva Oa) and on 79 equines in Tahiti Island. Data collected for each animal included the year of birth, location at the sampling date (municipality for New Caledonia and island for French Polynesia), origin (resident [i.e. born in New Caledonia or French Polynesia, respectively] or imported) and the date of importation when relevant. In New Caledonia, 40 horses were imported (29 from Australia, 4 from New Zealand and 7 from France) while in French Polynesia, 17 were imported (15 from New Zealand and 2 from New Caledonia).

### cELISA

Horse sera were screened using a cELISA test (ID Screen West Nile Competition ELISA kit, ID Vet, Montpellier, France). This ELISA uses plates pre-coated with the envelope (E) protein of WNV and measure the competition between antibodies present in the animal serum tested and a monoclonal anti-WNV.E antibody conjugated to horseradish peroxidase (HRP). 50μL of samples mixed with equal volume of dilution buffer were added to the wells coated with recombinant WNV.E protein during 90 min at room temperature (RT). The anti-E antibodies, if present, formed an antigen-antibody complex. 100μL of monoclonal anti-WNV.E antibody peroxidase (HRP) conjugate was then added to the wells during 30 minutes at room temperature (RT), possibly forming an antigen-conjugate-HRP complex. After addition of 100μL of substrate solution during 15 minutes at RT, coloration was stopped by the addition of 100μL of stop solution and micro-plates were read with a spectrophotometer at 450 nm wavelength. Results were validated if the mean value of the optical density (OD) of the negative control (NCOD) was > 0.7, and the mean value of the positive control OD (PCOD) was < 30% of the NCOD. Then, we computed the S/N percentage (S/N%): 100 * sample OD / NCOD. Samples showing a S/N% ≤ 40% were considered as positive. Those with 40% < S/N% ≤ 50% were considered as doubtful. Samples with S/N% > 50% were considered as negative. This test detects antibodies directed against the WNV envelope glycoprotein, but cross-reactions with mosquito-borne flaviviruses (USUV, JEV) and also more distant tick-borne flaviviruses (TBEV) have been reported [21–27].

WNV belongs to the JEV serocomplex, sharing 76% of the envelop aminoacid (aa) homology with JEV. Other serocomplexes include tick-borne encephalitis virus (TBEV), ZIKV and DENV1 sharing 35%, 51% and 46% aa homology respectively with the E protein of WNV. Furthermore, conserved epitopes beyond flaviviruses, are present at the surface of this protein. The cELISA test represents though, a convenient tool to detect antibodies directed against viruses belonging to the genus *Flavivirus* (WNV or other flaviviruses).

### Microsphere immunoassay (MIA)

A flavivirus microsphere immunoassay (MIA) was performed on positive cELISA samples with detection of flaviviruses that cause severe neuro-invasive infections in humans and horses. Briefly, the recombinant soluble ectodomain of the WNV envelope (E) glycoprotein (WNV.sE) and the E domain III (rEDIII) of WNV, JEV and tick-borne encephalitis virus (TBEV) containing virus-specific epitopes was kindly provided by Dr Philippe Desprès and were synthesized using the *Drosophila* S2 expression system [28]. Antigens produced were covalently bound to fluorescent beads following the protocol previously described in refs. [25, 28]. Diluted serum samples (1:100) were incubated with the beads for 1 h. A secondary biotinylated goat anti-horse IgG (dilution 1:500; Jackson Immuno Research Inc.) was then added. After an incubation of 45 min, the reaction was revealed with streptavidin R-phycoerythrin conjugate (SAPE; 1 μg/mL; Qiagen), diluted to 1:100. The median fluorescence intensity (MFI) of each microsphere set was quantified using a Bio-Plex 200 instrument (Bio-Rad Laboratories). The cutoffs of WNV.sE, WNV.EDIII, JEV.EDIII and TBEV.EDIII antigens were found to be 17, 54, 55 and 61, respectively, as described in ref. [28].

### Seroneutralization tests

Flaviviruses identified by MIA and all undetermined ELISA-positive flavivirus samples were further investigated using virus-specific microneutralization tests (MNT) against the main flaviviruses reported in the area where the sera were collected (i.e. JEV, WNV, ZIKV and DENV1). The World Organization for Animal Health (OIE) and the World Health Organization (WHO) recommend the use of the plaque reduction neutralization test (PRNT) with a threshold plaque reduction level of 90% (PRNT90) as the gold standard confirmatory assay [29, 30]. The MNT is a modification of the PRNT90. Its performance is comparable to the PRNT90 and allows a larger number of samples to be screened using cell microplates [31]. Neutralizing antibody titers were assessed in 96-well cell culture plates on Vero NK cells (kindly provided by Philippe Desprès) with the JEV genotype III Nakayama strain (GenBank accession no. EF571853), the WNV lineage 1 IS-98-ST1 strain (AF481864) following the protocol described in ref. [28]. Heat-inactivated sera, serially diluted (1/5 to 1/3645 by serial 1/3 dilutions) were mixed with an equal volume (50 μL) of DMEM containing 100 tissue culture infectious dose 50 (TCID50) of WNV or JEV strains. After incubation of the plates at 37°C for 1.5 h, 2x 10^4 Vero cells in 100 μL of DMEM were added to every well. Plates were incubated at 37°C for 3 days and presence of cytopathogenic effects (CPE) were observed under a light microscope. Results were validated if (i) CPE were absent in the mock infected control, (ii) CPE were observed in the infected control, (iii) virus titre was equal to the defined titre ±25% TCID50 per well, (iv) no protective effect was seen with the negative reference serum and (v) the positive reference serum protected Vero cells from infection. A serum was considered negative if CPE were observed at all the serum concentration tested. A serum was considered as positive, if it displayed protection (no CPE) at the 1/10 dilution; its titre was calculated as the inverse of the last dilution at which cells were protected.

MNT with the ZIKV strain/PF/2013 (GenBank accession no. KJ776791.2) [32] and DENV1, strain Djibouti/2000 were performed according to the following protocol described in ref. [33]:

Serial dilutions of heat inactivated serum samples (1/10 for the first dilution followed by serial half dilutions until 1/80) were mixed with an equal volume (50 μL) of DMEM containing 50 TCID50 of ZIKV or DENV1 strains. After incubating the mixtures at 37°C for 1 h, each virus-diluted serum sample (0.1 ml) was inoculated onto 96-well microtitre plates that had been seeded 24 h earlier with Vero NK cells, 10^5^ cells/ml. Plates were incubated at 37°C for 4 days (ZIKV) and 6 days (DENV1) and cytopathogenic effects were observed under a light microscope. The assay was validated as above and the serum was considered positive if cells were protected at the 1/20 serum dilution.

Due to serological cross-reactivity induced by viruses belonging to the Japanese encephalitis virus serocomplex (i.e. JEV and WNV), the MNT results were interpreted according to the following rules: (1) if the antibody titer was positive for only one flavivirus or if one titre was fourfold greater than the other, the serum was identified as containing antibodies against the virus displaying the highest positive serum dilution; (2) for differences in antibody titers less than fourfold, the virus identification could not be determined.

ZIKV does not belong to the dengue serocomplex [3]. For this reason and due to high endemicity of DENV1 in these areas and emergence of ZIKV at least 1.5 years before horse sampling, a serum was classified positive for both DENV1 and ZIKV in case of positive results against these two viruses and the corresponding animals were assumed to have been exposed to both viruses.

In cases of negative MNT results, but positive ELISA, the serum was classified as positive against an undetermined flavivirus.

### Statistical analysis

Seroprevalence rates were computed and compared according to the area (New Caledonia or French Polynesia), and for resident and imported horses, using Fisher’s exact tests. Doubtful cELISA test results were considered as negative when computing seroprevalences. The relationship between age and seropositivity was investigated using Student’s *t*-tests. All statistical analyses were performed using R vers. 3.2.3 [34].

## Results

### cELISA

Out of 163 sera from New Caledonia, 27 (16.6%, 95% confidence interval [CI]: 11.2%-23.1%) were positive, 3 (1.8%) doubtful, and 133 (81.6%) negative. Out of the 130 sera collected in French Polynesia, 40 (30.8%, 95% CI: 23.0%-39.5%), were positive, 5 (3.8%) doubtful and 85 (65.4%) negative (Table 1). The flavivirus-positive equines were located in different parts of New Caledonia and were located on Marquesas (8) and Tahiti (32) islands in French Polynesia.

**Table 1:** Global cELISA flavivirus results and DENV1, ZIKV, WNV, and JEV MNT confirmation on positive cELISA results in horses sampled in New Caledonia and French Polynesia.

**New** **Caledonia**	**Number** **of** **horses** **sampled**	**cELISA**			**MNT confirmation of positive cELISA results**						
		Negative Ig	Doubtful Ig	Positive Ig	DENV1	ZIKV	ZIKV and DENV1	WNV	JEV	WNV or JEV	Undetermined flavivirus
Resident horses	123	105	1	17	6	1	2	0	0	0	8
Imported horses	40	28	2	10	0	2	2	2	1	1	2
Total	163	133	3	27	6	3	4	2	1	1	10
**%**		**81.6**	**1.8**	**16.6**	3.7*	1.8*	2.5*	1.2*	0.6*	0.6*	6.1*
**French** **Polynesia**	**Number** **of** **horses** **sampled**	Negative Ig	Doubtful Ig	Positive Ig	DENV1	ZIKV	ZIKV and DENV1	WNV	JEV	WNV or JEV	Undetermined flavivirus
Resident horses	113	78	5	30	7	12	1	0	0	0	10
Imported horses	17	7	0	10	1	6	1	0	0	0	2
Total	130	85	5	40	8	18	2	0	0	0	12
**%**		**65.4**	**3.8**	**30.8**	6.2*	13.8*	1.5*	**0**	**0**	**0**	9.2*

* The percentage was based on total sample numbers (assuming all cELISA negative would also be negative by MNT)

Based on the cELISA results, the overall immunoglobulin (Ig)-anti-flavivirus seroprevalence was significantly lower in New Caledonia than in French Polynesia (Fisher’s exact test, p = 0.0005). This difference was observed in resident horses (Fisher’s exact test, p = 0.02) and in imported animals (Fisher’s exact test, p = 0.03).

### Microsphere immunoassay (MIA)

An in-house flavivirus multiplex assay was used to screen specific IgG antibodies against encephalitic flaviviruses among the 67 cELISA-positive sera from French Polynesia or New Caledonia. Based on the cut-offs defined in ref. [28], 91% (61/67) of cELISA positive sera were also positive to WNV.sE beads confirming the infection with a flavivirus. The WNV.sE based MIA was found to be less sensitive (Se) than ELISA for the detection of anti-flavivirus antibodies. Only 9.0% (6/67) and 6.0% (4/67) of sera were also positive for JEV and WNV, respectively. Therefore, more than 85.1% (57/67) were deemed infected with undetermined flaviviruses. To confirm cELISA and MIA results, an MNT against WNV and JEV was carried out at ANSES, and another MNT–against DENV1 and ZIKV–was done at the Armed Forces Biomedical Research Institute (Institut de recherche biomédicale des armées [IRBA]).

### Seroneutralization tests

#### Dengue virus serotype 1 serosurvey

In New Caledonia, 10 horses from six municipalities (titer range: 20- superior or equal to 80) were MNT DENV1-positive: 8 were resident horses and 2 had been imported. The corresponding DENV1 seroprevalence was 6.1% (10/163, 95% CI: 3.0%-11.0%).

In French Polynesia, eight resident and two imported horses were MNT DENV1-positive (titer range: 20- superior or equal to 80) resulting in 7.7% seroprevalence (10/130, 95% CI: 3.8%-13.7%) (Table 1). The positive resident MNT DENV1 horses were from Tahiti (5) and the Marquesas Islands (3).

**Fig 2:**
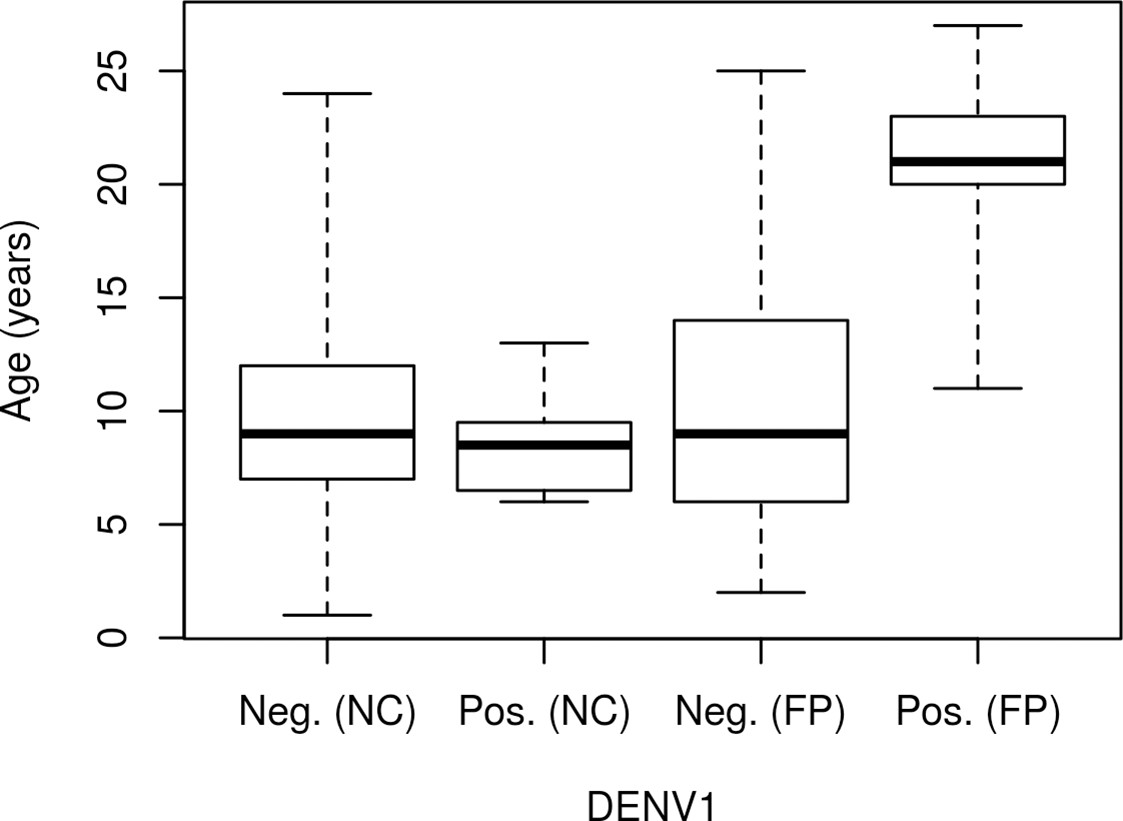
Box plot representing the distribution of DENV1-negative and -positive resident horses in New Caledonia (NC) and French Polynesia (FP) according to the age of the animal.

No significant difference in DENV1 seroprevalence was observed in resident horses between New Caledonia and French Polynesia. However, in resident animals from French Polynesia, seropositive horses were significantly older than seronegative animals (Student’s *t*-test, p = 0.02, Fig 2), but this difference was not observed in NC (Student’s *t*-test, p = 0.18, Fig 2).

#### Zika virus serosurvey

**Fig 3:**
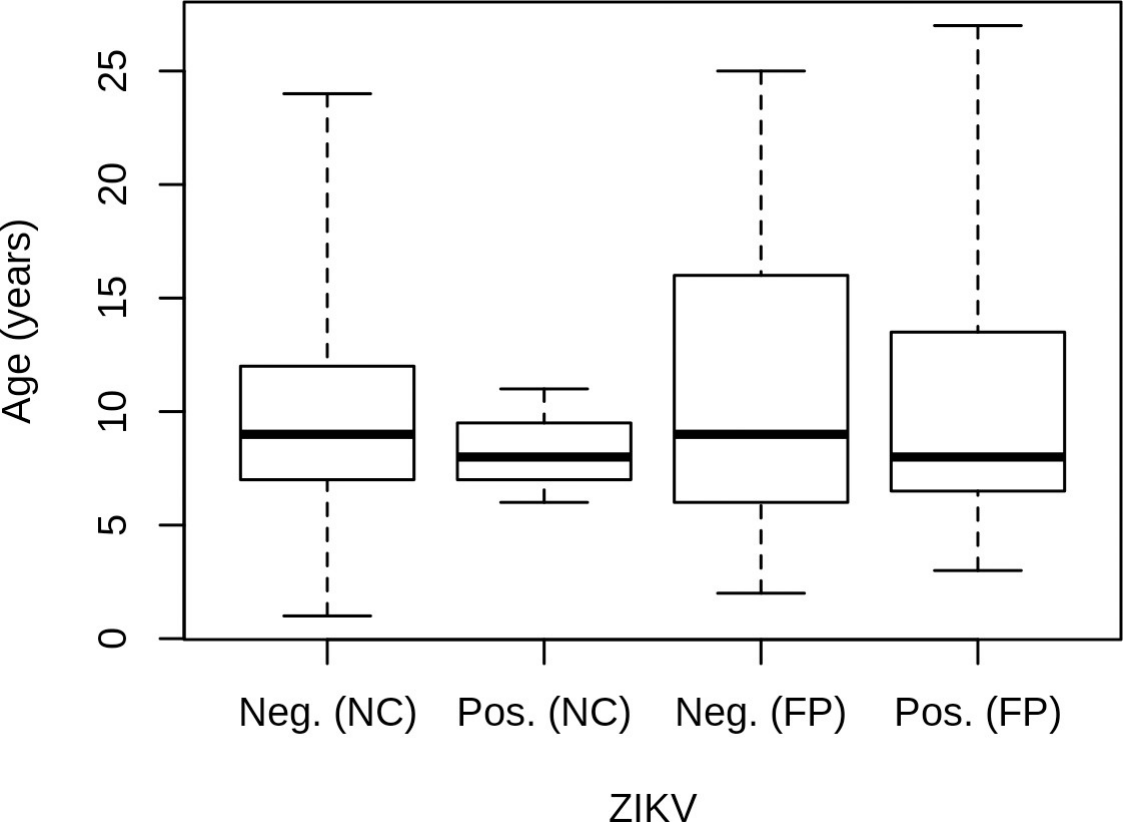
Box plot representing the distribution of ZIKV-negative and -positive resident horses found in New Caledonia (NC) and French Polynesia (FP) according to the age of the animal.

In New Caledonia, three resident and four imported horses from six municipalities were MNT ZIKV-positive (titer = 40). The corresponding seroprevalence was 4.3% (7/163, 95% CI: 1.7%-8.6%). Two resident and two imported horses also had a positive MNT result for both DENV1 and ZIKV (Table 2). In French Polynesia, the number of positives was higher, with 13 resident and 7 imported positive horses using MNT (titer range = 20-superior or equal to 80). The corresponding seroprevalence was 15.4% (20/130, 95% CI: 9.7%-22.8%). One resident and one imported horse also had a positive MNT result for DENV1 and ZIKV (Table 2). Most resident French Polynesia positives horses were from Tahiti (11 horses), but two resident horses were from Marquesas. Seroprevalence in resident horses was significantly higher in French Polynesia than in New Caledonia (Fisher’s exact test, p = 0.008). No significant association between age and ZIKV seropositivity was observed, neither in New Caledonia nor in French Polynesia (Fig 3).

**Table 2:** Results of DENV1 and ZIKV microneutralization tests on cELISA-positive samples. Microneutralization test results are expressed as negative or positive (i.e. titers ≥ 20).

New Caledonia	DENV1 ZIKV	Negative	20	40	≥ 80	**Total**
	Negative	14	1	4	1	**20**
	20	/	/	/	/	**0**
	40	3	2	2	/	**7**
	≥ 80	/	/	/	/	**0**
	**Total**	**17**	**3**	**6**	**1**	**27**
French Polynesia	DENV1 ZIKV	Negative	20	40	≥ 80	**Total**
	Negative	12	2	3	3	**20**
	20	7	/	/	/	**7**
	40	1	/	/	/	**1**
	≥ 80	10	1	1	/	**12**
	**Total**	**30**	**3**	**4**	**3**	**40**

#### Other flaviviruses

Using MNT, we identified four sera positive against WNV and/or JEV. Two of them were found positive against only WNV (titer range 30–90) and one against JEV (titer = 10). For the fourth animal, the infecting flavivirus could not be determined due to a difference less than fourfold, in neutralizing antibody titers obtained for WNV (titer = 30) and JEV (titer = 10).

Regarding the 67 sera tested, MIA was shown to be as sensitive (Se = 100%) but less specific (Sp = 97% [63/65] and 93.8% [61/66] on WNV and JEV respectively) than MNT (Table 3).

**Table 3:** Contingency table between MNT and MIA.

MNT MIA	WNV	JEV	Negative or undetermined	Total
WNV	2	0	2	4
JEV	0	1	5	6
Negative or undetermined	0	0	57*	57
**Total**	2	1	64	67

* The serum positive for WNV and JEV with a difference less than fourfold in neutralizing antibody titers was classified as undetermined

These four WNV and/or JEV positive sera were all sampled from horses imported from Australia between 2002 and 2009. Finally, we were not able to determine which flavivirus was responsible for the infection of 6.1% and 9.2% of the sampled horses in New Caledonia and French Polynesia, respectively (Table 1).

## Discussion

Our study based on three different and complementary serological methods allowed the detection and identification of flaviviruses responsible for seropositivity in horses sampled in New Caledonia and French Polynesia. Generic anti-flavivirus antibodies have been detected by cELISA in 67/293 horse sera and 91% (61/67) of them were also found reactive to the WNV.sE bead by MIA. These two independent methods corroborated the detection of anti-flavivirus antibodies in a significant proportion of the horse population [28].

Identification of the infecting flavivirus is challenging because most serological tests identifying past-exposure to flaviviruses through IgG detection, and in particular MIA and cELISA used in this study, are based on the envelop (E) antigen sharing flavivirus cross-reactive epitopes located in particular within the highly conserved fusion peptide in E.DII [35]. To improve the specificity of diagnostic assays, flavivirus positive sera were tested by MIA using WNV.EDIII, JEV.EDIII and TBEV.EDIII antigens which contain virus-specific-epitopes [36, 37] and by MNT against WNV and JEV. The results of the two methods were in accordance on three sera (two WNV positive sera and one JEV positive sera). Seven MIA samples were found positive against JEV (5 sera) and WNV (2 sera) and negative with JEV and WNV MNT respectively. Such discordant results could originate from a higher sensitivity or a lack of specificity of the flavivirus MIA technique. The fact that 4 out of the 7 WNV/JEV MIA positive and MNT negative sera were found ZIKV positive by MNT support the second hypothesis. Correspondingly, WNV.EDIII and JEV.EDIII share 74% of aa homology and a higher homology with ZIKV.EDIII (aa homology of 59% and 52% respectively) than with DENV1.EDIII (aa homology of 40% and 47% respectively) or TBEV.EDIII (aa homology of 29% and 36% respectively).

Based on the low percentage of horses found positive against WNV and JEV, the sera were tested by MNT against ZIKV and DENV1 at the IRBA laboratory. The standard operating procedures of the two MNT performed at ANSES (WNV and JEV) and IRBA (DENV1 and ZIKV) are comparable (with input virus 2-fold lower for ZIKV or DENV1 than for JEV and WNV and with positivity thresholds two times higher for ZIKV and DENV1 than for WNV and JEV), affording good specificity in flavivirus identification by MNT. Consequently ZIKV and DENV infections in horses can be established with high confidence in French Polynesia and New Caledonia.

In the Pacific Islands, mosquito-borne flaviviruses such as DENV or ZIKV are transmitted by *Aedes* mosquitoes with *Ae*. *aegypti* being the main competent vector present in French Polynesia and New Caledonia and *Ae*. *polyniensis* the secondary vector [1, 17]. No comparative competence studies in *Aedes* mosquitoes, between DENV and ZIKV local isolates, are available in South Pacific. However, sequential studies support higher dissemination and transmission rates of indigenous mosquito species for DENV1 only [38–40]. The presence of specific antibodies against DENV and ZIKV in horse populations suggests that, as referenced in the literature, the *Aedes* genus, known to have primate reservoir hosts, can opportunistically feed on other mammalian hosts. In Indonesia, antibodies against ZIKV have been detected in various vertebrate hosts such as ducks, goats, horses, bats and buffaloes [18]. Blood feeding studies of *Ae*. *aegypti* mosquitoes indicated that 99% and >85% engorged *Ae*. *aegypti* females fed on humans in Thailand and in islands of India respectively [41, 42]. One study also demonstrated that horses correspond to minor feeding hosts for *Ae*. *aegypti*, far below humans and dogs (≤0,8% vs 76–79% and 18–21% for humans and dogs respectively) [19]. Such a finding can help explaining differences in exposure levels of humans and horses to *Aedes*-borne viruses: 80% and 49% seroprevalence, for DENV1 and ZIKV respectively, in the 2014 French Polynesia human population in comparison with 7.7 and 15.4% in the French Polynesia equine population [43, 44]. However, if *Ae*. *albopictus*, with a more flexible feeding pattern, spread to South Pacific, horse exposure to DENV or ZIKV could be enhanced.

The South Pacific has faced successive outbreaks due to one of the four DENV serotypes [4, 6, 45]. DENV1 had been circulating for several years with epidemic periods in 2006–2007 in French Polynesia and 2008–2009 in New Caledonia before reemerging again in 2012–2013 in New Caledonia and French Polynesia [8, 46]. Accordingly, there were no significant differences in the DENV1 seropositivity rates in resident horses in New Caledonia (6.5%; 95% CI 2.1%-10.9%) and French Polynesia (7.1%; 95% CI 2.4%-11.8%). This result suggests similar circulation dynamics for DENV1 in both areas. However, interestingly, the differences between New Caledonia and French Polynesia in recent flavivirus circulation correlate with the different seroprevalence levels observed in our study. This study shows that the age of the horse was associated with DENV1 seropositivity in French Polynesia, but not in New Caledonia. In 2012–2013, New Caledonia experienced an important outbreak with more than 10,000 human cases reported [1, 46]. This large wave of DENV1 infections may have randomly affected the different age classes in the New Caledonia equine population. Conversely, in French Polynesia, although DENV1 reemerged in 2013–2017, the age-seroprevalence correlation suggests that the positive horses detected in our study likely result from older DENV1 circulation events, because the seroprevalence rate is expected to increase with increased duration of exposure to the virus [8].

The situation is clearly distinct for ZIKV. The first Pacific ZIKV outbreak occurred in 2007 on Yap Island before its reemergence in French Polynesia in October 2013 [17, 47]. This new introduction resulted in an explosive outbreak in French Polynesia and spread throughout the South Pacific to New Caledonia [48]. At the end of the outbreaks, French Polynesia reported 8723 suspected human cases and more than 30,000 estimated clinical visits due to ZIKV, whereas 11,000 estimated cases and 1,400 confirmed cases had been reported in New Caledonia [1]. Similarly, our study demonstrated a higher seroprevalence rate in French Polynesia resident horses than in New Caledonia. The absence of a significant age effect in French Polynesia suggests that horse population was naïve before this new emergence. In New Caledonia, due to the low number of seropositive resident horses (3 animals or 1 if the 2 sera positive for both ZIKV and DENV1 are not taken into account) the link between seropositivity and age is more difficult to assess.

Finally, the low seroprevalence of JEV and WNV flaviviruses in equines (2.4% in NC and no positive samples in FP) suggests the absence of past active circulation of these viruses in New Caledonia and French Polynesia, which is consistent with levels of JEV and WNV seroprevalence (< 1.5%) among blood donors in French Polynesia in 2011–2013, for example [16]. The positive results for WNV or JEV obtained for four New Caledonia horses native to Australia may attest to past vaccination or exposure to these viruses before their importation in New Caledonia and French Polynesia.

We did not identify which flavivirus was involved in the infection of 6.1% and 9.2% of cases in New Caledonia and French Polynesia, respectively. The lower sensitivity of MNT compared to cELISA and/or the circulation of other flaviviruses, particularly the three other DENV serotypes, may explain why no virus could be identified [21]. Several other flaviviruses have been specified in Australasia, but not described in New Caledonia nor in Polynesia. In Australia or Papua New Guinea, Murray Valley Encephalitis Virus, a *Culex*-borne flavivirus classified in the JEV serocomplex, the Kokobera virus (and its Stratford subtype) associated with polyarticular disease in humans also found in the *Culex*-group but ranking in a separate serocomplex have been described. Finally two *Aedes*-borne flaviviruses (Sepik/Fitzroy River virus, closely related to the African Wesselsbron virus, and the Edge Hill virus) have been regularly reported in recent years. Animals infected with such flaviviruses would be expected to generate false positive reactions with the ID Vet WNV cELISA kit, while not reacting in specific WNV/JEV (apart from MVEV classified in the JEV serocomplex), or ZIKV/DENV MNT, as inferred from the low genetic and antigenic relationships between Kokobera and WNV/JEV and between Sepik River virus, Edge Hill virus, DENV and ZIKV [49–52].

In conclusion, our study clearly shows that horses can be infected by *Aedes* mosquito-borne viruses such as DENV1 or ZIKV, which are known to have a primate reservoir. The seroprevalence rate for these viruses associated with serological cross-reactions involving related flaviviruses challenges the diagnosis of flaviviruses in horses [21].

Although the DENV1 and ZIKV seropositivity rates are clearly lower in horse populations than in the general human population (7.7 and 15.4% of antibodies against DENV1 and ZIKV respectively, in the French Polynesia equine population compared to 80% and 49%, respectively, in the 2014 French Polynesia human population) [43, 44], our study emphasizes the need to acquire additional data regarding domestic animals in ZIKV and DENV epidemiological cycles.
